# The clinical features, outcomes and genetic characteristics of hypertrophic cardiomyopathy patients with severe right ventricular hypertrophy

**DOI:** 10.1371/journal.pone.0174118

**Published:** 2017-03-21

**Authors:** Xiying Guo, Chaomei Fan, Lei Tian, Yanling Liu, Hongyue Wang, Shihua Zhao, Fujian Duan, Xiuling Zhang, Xing Zhao, Fengqi Wang, Hongguang Zhu, Aiqing Lin, Xia Wu, Yishi Li

**Affiliations:** 1 Key Laboratory of Clinical Trial Research in Cardiovascular Drugs, Ministry of Health, Fuwai Hospital, Chinese Academy of Medical Sciences and Peking Union Medical College, Beijing, China; 2 Department of Ultrasound, Fuwai Hospital, Beijing, China; 3 Department of Pathology, Fuwai Hospital, Beijing, China; 4 Department of Radiology, Fuwai Hospital, Beijing, China; 5 Department of Cardiology, Anqiu Chinese Medicine Hospital, Anqiu, China; 6 Department of Cardiology, Heze Zone Central Hospital, Heze, China; 7 Department of Cardiology, Zhecheng People’s Hospital, Zhecheng, China; 8 Department of Cardiology, The Harbor Hospital of Yantai, Yantai, China; 9 Department of Cardiology, Muping District Chinese Medicine Hospital of Yantai, Yantai, China; 10 Department of Cardiology, Laiwu Central Hospital, Laiwu, China; Mayo Clinic, UNITED STATES

## Abstract

**Introduction:**

Severe right ventricular hypertrophy (SRVH) is a rare phenotype in hypertrophic cardiomyopathy (HCM) for which limited information is available. This study was undertaken to investigate the clinical, prognostic and genetic characteristics of HCM patients with SRVH.

**Methods:**

HCM with SRVH was defined as HCM with a maximum right ventricular wall thickness ≥10 mm. Whole-genome sequencing (WGS) was performed in HCM patients with SRVH. Multivariate Cox proportional hazards regression models were used to identify risk factors for cardiac death and events in HCM with SRVH. Patients with apical hypertrophic cardiomyopathy (ApHCM) were selected as a comparison group. The clinical features and outcomes of 34 HCM patients with SRVH and 273 ApHCM patients were compared.

**Results:**

Compared with the ApHCM group, the HCM with SRVH group included younger patients and a higher proportion of female patients and also displayed higher cardiovascular morbidity and mortality. The multivariate Cox proportional hazards regression models identified 2 independent predictors of cardiovascular death in HCM patients with SRVH, a New York Heart Association class ≥III (hazard ratio [HR] = 8.7, 95% confidence interval (CI): 1.43-52.87, *p* = 0.019) and an age at the time of HCM diagnosis ≤18 (HR = 5.5, 95% CI: 1.24-28.36, *p* = 0.026). Among the 11 HCM patients with SRVH who underwent WGS, 10 (90.9%) were identified as carriers of at least one specific sarcomere gene mutation. *MYH7* and *TTN* mutations were the most common sarcomere mutations noted in this study. Two or more HCM-related gene mutations were observed in 9 (82%) patients, and mutations in either other cardiomyopathy-related genes or ion-channel disease-related genes were found in 8 (73%) patients.

**Conclusions:**

HCM patients with SRVH were characterized by poor clinical outcomes and the presentation of multiple gene mutations.

## Introduction

Hypertrophic cardiomyopathy (HCM) is the primary disease affecting the cardiac muscle and is characterized by heterogeneous genetic, morphological, functional, and clinical features. Left ventricular hypertrophy is the most characteristic feature of HCM [1–2]. Severe right ventricular hypertrophy (SRVH) is a relatively rare subtype of HCM in which myocardial hypertrophy primarily affects the right ventricle, which is generally ignored in HCM in clinical practice. Consequently, the anatomic, genetic, clinical, and prognostic characteristics of patients with SRVH and the clinical relevance of these characteristics have not been described well in the literature. Apical hypertrophic cardiomyopathy (ApHCM) is also a relatively rare variant of HCM and has become a well-established phenotype as a result of its investigation in previous studies; however, ApHCM still follows a variable clinical course [3–6]. Whole-genome sequencing (WGS) can be used to study the genetic basis of HCM. The aims of this study were to investigate the clinical, genetic and prognostic characteristics of HCM patients with SRVH and to compare these data with those of patients with ApHCM.

## Materials and methods

### Study patients

Subjects were selected from among 2650 HCM patients treated at Fuwai Hospital, Anqiu Chinese Medicine Hospital, Heze Zone Central Hospital, Zhecheng People’s Hospital, Muping District Chinese Medicine Hospital of Yantai, and Laiwu Central Hospital from 1996 to 2013. A total of 34 HCM patients with SRVH and 273 ApHCM patients were enrolled in the study, which was approved by the research ethics boards of Fuwai Hospital, Anqiu Chinese Medicine Hospital, Heze Zone Central Hospital, Zhecheng People’s Hospital, Muping District Chinese Medicine Hospital of Yantai, and Laiwu Central Hospital. Because this was not a clinical trial, patients provided verbal informed consent for the use of their clinical information in this study. Their names and personal information were kept confidential. The data for each patient were not recorded in the HCM database unless the corresponding patient agreed to participate in the study. This consent procedure was approved by all ethics committees involved in the study.

### Diagnostic criteria

Unified criteria for the diagnosis of right ventricular (RV) wall hypertrophy in the context of HCM have not yet been defined. In our study, HCM was defined as a wall thickness ≥15 mm in adults or the equivalent relative to body surface area in children during end-diastole in the absence of any other cause [7]. Therefore, SRVH was characterized by an RV anterior, free or apical wall thickness ≥10 mm during end-diastole [8]. RV outflow tract obstruction (RVOTO) was defined as an RV outflow tract pressure gradient exceeding 25 mmHg under resting conditions [9]. Patients with pulmonary hypertension, pulmonary stenosis or other congenital heart diseases were excluded from the study [8]. The diagnosis of ApHCM was based on the presence of asymmetric hypertrophy that was confined predominantly to the left ventricular apex and was characterized by a maximal apical wall thickness ≥15 mm and a maximal apical-to-posterior wall thickness ratio ≥1.5.

Syncope, atrial fibrillation, non-sustained ventricular tachycardia, progressive heart failure with an increase in New York Heart Association (NYHA) class ≥1, and embolic stroke were considered cardiovascular morbidities [8]. Data regarding patient survival and clinical status were obtained either via detailed interviews or reviews of medical records. The primary clinical endpoints of this study were sudden cardiac death (SCD), heart failure-related death, stroke-related death, aborted cardiac arrest and the appropriate discharge of an implantable cardioverter defibrillator for ventricular fibrillation [10].

### Echocardiography

The RV free wall was measured at the level of the chordae tendineae in the subcostal 4-chamber view during end-diastole [11]. The RV anterior wall was evaluated in the RV inflow tract view, and the moderator band, free wall and RV apex were observed in the RV-focused apical four-chamber view [12]. Increased RV wall thickness was defined as a thickness ≥2 SDs greater than that of the control group, and severe wall thickness was defined as a thickness ≥4 SDs (approximately 10 mm) greater than that of the control group [8]. Doppler echocardiography was used to locate the site of the obstruction. If the peak flow velocity exceeded 2.5 m/s, which is equivalent to a peak pressure gradient greater than 25 mmHg under resting conditions, the patient was diagnosed with RVOTO [9,13]. A high flow velocity signal was detected via color flow mapping, confirming the obstruction location. Maximal apical wall thickness was evaluated using apical 2-chamber and apical 4-chamber views at end-diastole, and the mean values of these parameters were measured [14].

### Cardiac magnetic resonance (CMR) imaging

CMR imaging was performed with a 1.5-T system (MAGNETOM, Avanto, Siemens, Erlangen, Germany). Breath-hold electrocardiography-gated cine steady-state free precession images were acquired at the RV outflow tract, as well as in short-axis slices and in standard 2- and 4-chamber long-axis orientations [8]. A delayed gadolinium enhancement protocol was utilized following the intravenous administration of 0.2 mmol/kg gadolinium diethylenetriamine penta-acetic acid (Magnevist, Schering, Berlin, Germany), and images were obtained in the same views as the cine images.

### WGS of HCM patients with SRVH

WGS was performed in the 11 HCM patients with SRVH whose blood samples were available. Genomic DNA was isolated from those blood samples. The concentrations and size distributions of the DNA libraries were determined using an Agilent Bioanalyzer DNA 1000 chip, and the DNA libraries were sequenced using Illumina HiSeq X (Illumina, San Diego, CA), according to the manufacturer’s instructions for paired-end 150-bp reads. Following a quality control evaluation of the raw data, we mapped valid sequencing data to the reference genome (UCSC hg19) to obtain the original mapping results. SAMtools was used for variant calling and the identification of single nucleotide polymorphisms and indels. Control-FREEC was utilized for copy number variation detection, and Crest was utilized for the detection of structure variant information in variant calling.

ANNOVAR was utilized to annotate the variant call format obtained. The DbSNP, the 1000 Genome Project, the National Heart, Lung and Blood Institute (NHLBI) Exome Sequencing Project Database (ESP 6500), the International HapMap Project and the Pan Cardiomyopathy databases were utilized. In addition to the known pathogenic mutations that are present in these publicly available gene databases, possible pathogenic gene mutations should have been present within the cardiomyopathy/channelopathy-associated gene subset. Moreover, these possible pathogenic mutations must have been absent in a large panel of ethnically matched controls in these publicly available gene databases. Prediction of in silico pathogenicity for novel missense variants was performed using SIFT, Polyphen2, Mutation Taster, LRT and MetaLR prediction software. A variant was predicted to be pathogenic if classified as "damaging" by SIFT or either "possibly damaging" or "most likely damaging" by Polyphen2 [15].

Variants that were identified by WGS were validated using Sanger sequencing on an ABI3130XL Genetic Analyzer (Applied Biosystems, Foster City, CA, USA) to avoid false-positive high-throughput sequencing results.

### Follow-ups

Prospective clinical follow-ups were conducted for both groups of patients. Fuwai Hospital was responsible for following up with all 307 patients. Telephone calls and letters were the major patient follow-up methods utilized in this study. Detailed information was obtained, including information regarding each patient’s current situation (e.g., symptoms and NYHA classes), whether and when cardiovascular death or events had occurred, and any current treatments that patients were receiving.

### Statistical analysis

Continuous variables are presented as means ± standard deviations, and non-continuous variables are expressed as proportions. Survival estimates were calculated using the Kaplan-Meier method and the log-rank test. The annual event rate was calculated as the number of adverse clinical events divided by the average follow-up period in years. The risk factors for cardiac death and cardiac events and the 95% confidence intervals (CIs) were determined via univariate and multivariate Cox proportional hazards regression models. Data pertaining to baseline patient clinical characteristics, including data pertaining to sex, age, family history of either HCM or SCD, NYHA functional class and echocardiographic parameters, were analyzed via univariate Cox regression analysis. Variables demonstrating statistical significance in the univariate analysis (*p*<0.05) were subsequently entered into the multivariate Cox proportional hazards models. For all tests, *p*<0.05 was considered statistically significant. Analyses were performed with SAS software, version 9.2 (SAS, Institute, Cary, NC).

## Results

### Baseline clinical characteristics

Among the 2,650 HCM patients treated between 1996 and 2013, 34 HCM patients with SRVH were identified, for a prevalence of 1.3%. Compared with ApHCM patients, SRVH patients were more likely to be female, were much younger, and showed more symptoms at their initial evaluation. For HCM patients with SRVH, chest tightness was the most common presenting symptom, although edema was also common (n = 6, 17.6%). Eight patients exhibited decreased exercise tolerance and symptoms consistent with NYHA class III status. Initial analysis of patient electrocardiogram (ECG) recordings revealed that ST-T changes were the most common ECG abnormality (n = 16, 47.1%). Premature ventricular beats and atrial tachycardia (n = 11, 32.4%) were the two most frequent arrhythmias noted on patient Holter results. The baseline clinical characteristics of the patients of the two phenotypes are summarized in **Table 1,** as are the treatments that these patients received.

**Table 1:** The baseline clinical characteristics of HCM patients with SRVH and ApHCM patients.

Characteristics	HCM patients with SRVH	ApHCM patients	*p* value
	(n = 34)	(n = 273)	
**Basic characteristics**			
Female	19 (55.9)	70 (25.6)	0.0002
Age at diagnosis	30.5±15.7	50.8±12.6	<.0001
Chest pain	6 (17.6%)	82 (30.4%)	0.1319
Chest tightness	25 (73.5%)	161 (58.7%)	0.1015
Dyspnea	14 (41.2%)	25 (9.2%)	<.0001
Palpitation	20 (58.8%)	69 (25.4%)	<.0001
Pre-syncope	9 (26.5%)	3 (1.2%)	<.0001
NYHA class I/II	26 (76.5%)	265 (93.8%)	0.3000
NYHA class III/VI	8 (23.5%)	17 (6.2%)	0.3000
FH HCM	11 (32.4%)	15 (5.5%)	<.0001
NT-proBNP, pg/ml	2645±1170	921±711	<.0001
Systolic blood pressure, mmHg	118±20	127±11	0.006
Diastolic blood pressure, mmHg	69±8	78±9	0.0001
Heart rate, bpm	69±9	70±10	0.66
ALT, U/L	30.0±20.7	25.9±16.4	0.52
AST, U/L	29.6±17.7	21.4±6.4	0.07
TG, mmol/L	1.2±0.7	2.0±1.9	0.26
TC, mmol/L	4.5±0.9	4.3±1.5	0.86
Glucose, mmol/L	5.2±0.9	5.3±1.0	1.76
Serum creatinine,nmol/L	76.8±22.5	84.8±29.0	0.43
**Echocardiography**			
LAD, mm	40±8.7	37.2±6.2	0.0800
LVEDD, mm	41.9±7.9	47.4±5.4	0.0003
IVST, mm	18.7±4.4	14.5±7.7	<.0001
LVPWT, mm	10.9±2.1	10.0±9.7	0.0300
LVEF, %	62.3±13.1	66.5±9.2	0.1270
**LGE**	17 (81%)	15 (10.7)	<.0001
**Treatment**			
β-blockers or calcium-channel blockers	34 (100%)	259 (94.9%)	—
Invasive procedures	6 (17.6%)	0	—

The data are presented as n (%) or the means ± SDs.

ALT, alanine aminotransferase; AST, aspartate aminotransferase; ApHCM, apical hypertrophic cardiomyopathy; IVST, interventricular septum thickness; LAD, left atrial diameter; LVEDD, left ventricular end-diastolic diameter; LVPWT, left ventricular posterior wall thickness; LVEF, left ventricular ejection fraction; LGE, late gadolinium enhancement; NYHA, New York Heart Association; NT-proBNP, N-terminal pro-brain natriuretic peptide; SRVH, severe right ventricular hypertrophy; TC, total cholesterol; TG, total triglycerides.

### Echocardiography and CMR

**Fig 1:**
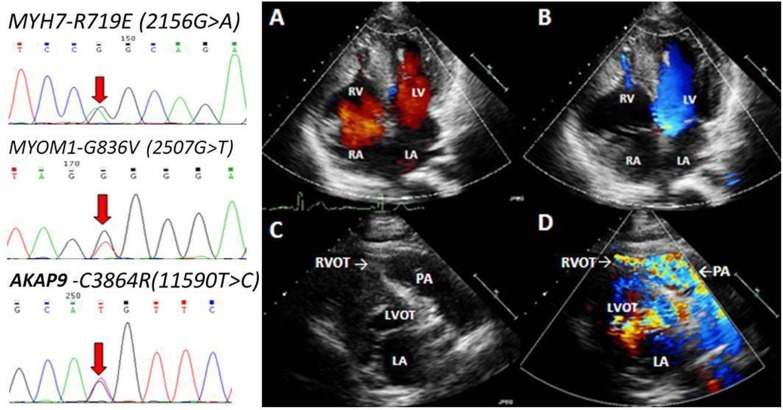
Transthoracic echocardiography and gene sequencing. An apical 4-chamber view of an 18-year-old patient with HCM demonstrating pronounced hypertrophy of the RV free wall during end-diastole (A) and RV cavity obliteration in the mid-ventricular region, as well as in the apical cavity (B). (C) Modified parasternal short-axis views with color Doppler flow mapping demonstrating striking hypertrophy of the RV anterior wall and narrowing of the RV outflow tract. (D) Color Doppler mapping demonstrating turbulent flow from the RV outflow tract to the PA (arrow). In the left panels, the red arrows indicate the double-peak at the site of the identified mutations. LA: left atrium; LV: left ventricle; LVOT: left ventricular outflow tract; PA: pulmonary artery; RA: right atrium; RV: right ventricle.

The echocardiographic variables of all patients at baseline are included in **Table 1**. Of the HCM patients with SRVH, 11 (32.4%) exhibited RVOTO at rest, with a peak pressure gradient of 52 mmHg, which increased slightly following an exercise provocation test. The average maximum RV wall thickness was 13.5±2.9 mm (10-22 mm, **Fig 1**). Compared with the ApHCM patients, HCM patients with SRVH presented with smaller left ventricular cavities during diastole and thicker interventricular septa and left ventricular posterior walls.

**Fig 2:**
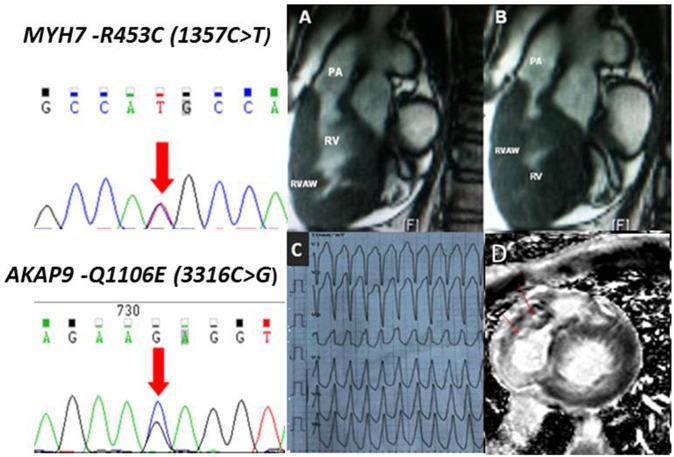
CMR and gene sequencing. CMR images from a 25-year-old HCM patient with SRVH. RV outflow tract long-axis views during end-diastole (A) and end-systole (B) demonstrating remarkable thickening of the RV anterior wall and apical region with obstruction. A Holter recording of a non-sustained ventricular tachycardia attack. RV short-axis view (D) demonstrating extreme thickening of the RV free wall and regional transmural LGE of the RV anterior and free walls (red arrow). In the left panels, the red arrows indicate the double-peak at the site of the identified mutations. LA: left atrium; LV: left ventricle; LVOT: left ventricular outflow tract; PA: pulmonary artery; RV: right ventricle; RVAW: RV anterior wall.

Twenty-one HCM patients (61.8%) with SRVH and 140 ApHCM patients (51.2%) underwent CMR. Narrowing of the RV outflow tract due to protrusion of the septum and a hypertrophic RV free wall were clearly observed, and the site of obstruction could be located precisely in 8 patients. Seventeen HCM patients with SRVH also exhibited detectable late gadolinium enhancement (LGE) of their hypertrophic RV wall, a finding suggestive of interstitial fibrosis and ventricular remodeling (**Fig 2**). Moreover, LGE was observed more frequently in HCM patients with SRVH than in patients with ApHCM (81.0% vs 10.7%; p<0.001).

### Follow-up

**Fig 3:**
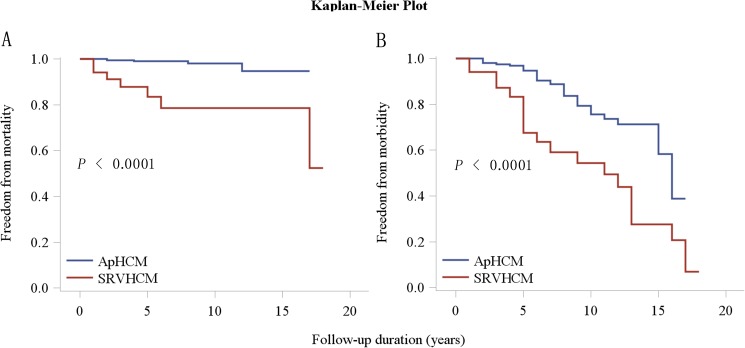
Kaplan-Meier estimates of the probability of cardiovascular survival (A) and freedom from cardiovascular morbidity (B) in HCM patients with SRVH and ApHCM patients (log-rank test for trend, *p*<0.001). ApHCM: apical hypertrophic cardiomyopathy; SRVH: severe right ventricular hypertrophy.

The mean follow-up times for HCM patients with SRVH and ApHCM patients were 6.9±5.3 and 7.9±3.5 years, respectively. The follow-up rate for HCM patients with SRVH was 100%. A total of 7 patients with SRVH died (4 patients suffered SCDs, and 3 suffered heart failure-related deaths), and the overall cardiovascular mortality rate was 20.6%. Approximately 61.8% of patients had more than one cardiovascular event. Of the patients with ApHCM, 262 were followed up. Thus, the follow-up rate was 96.0%. Four patients (1.53%, 2 patients who suffered SCDs and 2 patients who suffered heart failure-related deaths) and 47 (17.9%) patients experienced cardiovascular mortality and morbidity, respectively. Cardiovascular events were much more frequent in HCM patients with SRVH than in patients with ApHCM (**Table 2**). Log-rank analysis showed that cardiovascular survival and event-free survival were significantly lower in HCM patients with SRVH than in ApHCM patients (**Fig 3**).

**Table 2:** Clinical outcomes of HCM patients with SRVH and ApHCM patients during the follow-up period.

Characteristics	HCM patients with SRVH	ApHCM patients	*p* value
	(n = 34)	(n = 262)	
Years of follow-up	6.9±5.3	7.9±3.5	0.2590
**Cardiovascular mortality**	7 (20.6%)	4 (1.54%)	<.0001
Heart failure	3 (8.9%)	2 (0.78%)	0.0127
Sudden cardiac death	4 (11.8%)	2 (0.78%)	0.0003
**Cardiovascular morbidity**	21 (61.8%)	47 (19.7%)	<.0001
Thromboembolic events	2 (5.9%)	3 (1.2%)	0.2000
Syncope	8 (23.5%)	21 (7.7%)	0.0077
Atrial fibrillation	6 (17.6%)	28 (10.9%)	0.3904
Non-sustained ventricular tachycardia	6 (17.6%)	6 (2.4%)	0.0002
Myocardial infarction	0	2 (0.78%)	0.7780
Progressive heart failure	11 (32.4%)	15 (5.8%)	<.0001

The data are presented as n (%) or as the means ± SDs.

For HCM patients with SRVH, multivariate Cox regression analysis identified 2 independent predictors of cardiovascular death, an NYHA class of III or worse (hazard ratio, HR = 8.68, 95% CI: 1.43-52.87, *p* = 0.019) and an age ≤18 (HR = 5.45, 95% CI: 1.24-28.36, *p* = 0.026). An age ≤18 was also associated with a 4-fold increased risk of a cardiovascular events (95% CI: 1.43-12.93, *p* = 0.009). No significant differences in cardiovascular mortality and morbidity were detected between patients with and without RVOTO.

### Histological examination of HCM patients with SRVH

**Fig 4:**
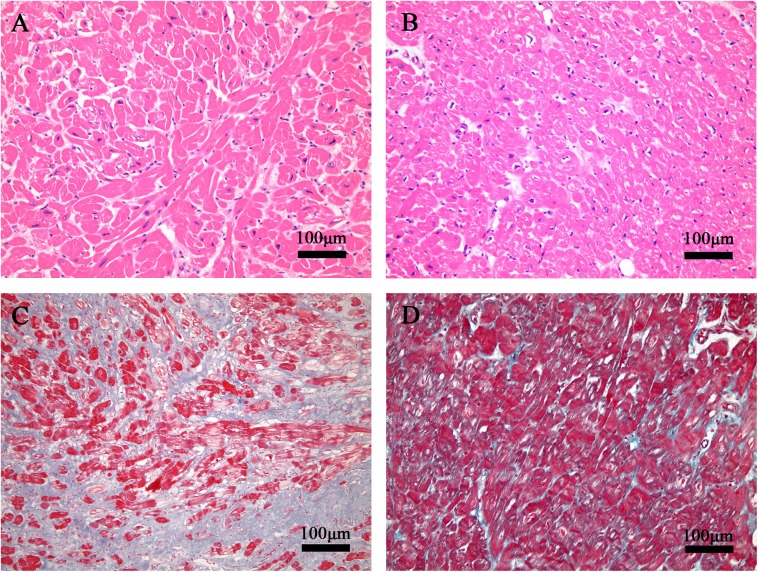
Histological findings. (A) Histological section of the RV free wall of an HCM patient with SRVH showing cardiomyocyte enlargement and disarray (hematoxylin-eosin staining, 200×) and (B) extensive interstitial fibrosis (Masson’s trichrome staining, 200×). (C) Histological section of the RV free wall of a patient with a double-chambered right ventricle demonstrating cardiomyocyte hypertrophy without disarray (D) and without interstitial fibrosis (E).

Myectomy specimens from the RV anterior and free walls were taken from two patients who underwent myocardial myectomy for RVOTO. Subsequent histological examinations revealed disorganized and abnormally enlarged cardiomyocytes, as well as interstitial fibrosis and scarring. These findings could indicate that this abnormal enlargement is different from that caused by pressure load (**Fig 4**).

### WGS results for HCM patients with SRVH

Following filtering (**S1 Fig**), we selected distinct rare nonsynonymous exonic or splice-site variants as candidates for further analysis. Ten patients (90.9%) carried at least one variant of the sarcomere genes that are associated with HCM. A total of 27 sarcomere gene mutations were identified, 23 of which were de novo mutations (**Table 3**). One patient carried only mutations associated with arrhythmogenic RV cardiomyopathy (ARVC) and dilated cardiomyopathy. Among all the sarcomere gene mutations noted in this study, mutations in the TTN gene (9 mutations) were the most common, followed by mutations in MYH7 (6 mutations) and MYBPC3 (4 mutations). Five (45.5%) patients exhibited dilated cardiomyopathy gene mutations, and 3 (27.3%) patients exhibited mutations associated with ARVC and ion-channel diseases. Nine (81.8%) patients exhibited two or more mutations involving HCM-related genes. Eight (72.7%) patients exhibited mutations related to other cardiomyopathies or ion-channel diseases (**Table 3**).

**Table 3:** A summary of the mutations affecting HCM patients with SRVH.

Case	Age at diagnosis	Sex	SCD risk factors	Arrhyt-hmia	NYHA class	RVWT (mm)	Sarcomere mutations	Non-sarcomere mutations	Prognosis
1	27	M	0		Ⅰ	13	MYH7-478N ^d^		
							TTN-S3872fs		
							TTN-S6350A ^d^		
							TTN-P10663R ^d^		
							ACTN2-P32fs		
2	43	M	0	AF	Ⅱ	14.9	MYH7-G708A ^d^	DSC2-S574R ^d^	Progressive NYHA
							MYBPC3-E334K ^d^	LAMA4-L1040P ^d^	Embolic
							TTN-E23808K ^d^	SYNE2-W5861C ^d^	
							ACTN2-P32fs		
3	57	F	2	NSVT	Ⅲ	11.5	MYH7-R652T^d^		Syncope
				AF			MYH6-A12V ^d^		
							TTN-E44L ^d^		
4	10	F	2		Ⅱ	13	TTN-I30627V ^d^	SYNE2-E6435K ^d^	Syncope
							TTN-E8694K^d^		
							MYBPC3-D248fs		
5	16	F	1	PVC	Ⅱ	12	MYBPC3-D610N*	DSP-M1fs	Syncope
							ACTN2-P32fs	RYR2-V1588L^d^	Progressive NYHA
							TTN-V20996F^d^		Ventricular-dilatation
6	28	F	1	PVC	Ⅰ	15	MYH7-R723C*		
							MYBPC3-E334K ^p^		
7	13	F	2		Ⅱ	12	MYH7-R719E*	AKAP9-C3864R ^p^	Syncope
							MYOM1-G836V ^d^		
8	61	F	3	NSVT	Ⅲ	13.8	TTN-D25178N ^d^	TRIM63-P113A ^d^	Progressive NYHA
				AF			TNNI3-R157C ^d^		Syncope
9	32	F	0		Ⅱ	14.1	MYL2-I35V ^p^	DSP-M1fs	Progressive NYHA
							ACTN2-P32fs	SCN5A-P627L ^d^	
10	18	F	2	NSVT	Ⅱ	14.3	MYH7-R453C*	AKAP9-Q1106E ^d^	Syncope
11	25	F	0	PVC	Ⅱ	11.6		SCN5A-S1902fs	
								CACNA1C-S1318F ^t^	
								DSG2-T236A ^t^	

AF, atrial fibrillation; NSVT, non-sustained ventricular tachycardia; NYHA, New York Heart Association; PVC, premature ventricular contraction; RVWT, right ventricular wall thickness; SCD, sudden cardiac death; SRVH, severe right ventricular hypertrophy.

*, HCM pathogenic gene.

d, damaged mutation predicted by SIFT or Polyphen2.

p, possible damaged mutation predicted by SIFT or Polyphen2.

t, tolerant mutation predicted by SIFT or Polyphen2.

SIFT, http://www.sift.jcvi.org/; Polyphen2, http://genetics.bwh.harvard.edu./pph2/.

## Discussion

Pathological changes of the right ventricle are often considered “secondary” to changes of the left ventricle. Moreover, the complex geometry of the right ventricle makes it difficult to measure. However, the right ventricle plays an important role in cardiac pathophysiology and is an independent predictor of death secondary to heart failure, as well as SCD [16]. We conducted WGS in 11 HCM patients with SRVH and identified novel mutations for HCM that may contribute to its diagnosis.

### Diagnostic measurements

Two-dimensional echocardiography is the most commonly used method for diagnosing HCM, but accurately evaluating the right ventricle is difficult [17]. Diagnostic standards for RV hypertrophy in the context of HCM are lacking. McKenna et al. defined RV hypertrophy as mild (6-8 mm), moderate (9-12 mm), or severe (>12 mm) [18–19], and Martin et al. defined increased RV wall thickness as a thickness ≥2 SDs and extreme wall thickness as a thickness ≥4 SDs (approximately 10 mm) greater than the mean thickness of the controls [8]. Shimizu et al. used 16 mmHg as the standard for RVOTO in HCM, although 25 mmHg is more widely used to diagnose RVOTO secondary to congenital or functional abnormalities. The absence of diagnostic criteria for RV diseases increases the difficulty of making accurate diagnoses. CMR may be a superior method for evaluating the morphology of the right ventricle and may enable clinicians to both locate hypertrophic regions and accurately evaluate wall thickness. In both infants and children, RV hypertrophy and RVOTO are usually caused by abnormalities in anatomy and growth, such as congenital subpulmonary infundibular stenosis, which may be distinguished from other conditions via CMR [20].

### Outcomes

HCM patients with SRVH were younger and more likely to be female than ApHCM patients and the total population of HCM patients in China (nationally, the average age of affected patients is 50 years, and 29% of affected patients are female) [21].The cardiovascular mortality rate was 20.6%, with an annual cardiovascular mortality rate of 3.0%, which was significantly greater than the annual mortality rate of 1.4% observed in the general HCM population and the rate of 0.2% observed in ApHCM patients. SCD was the most frequently observed cause of death, a phenomenon that may be attributed to the increased incidence of ventricular tachycardia among patients with RV hypertrophy [19].

Patients with RVOTO did not differ significantly from patients without RVOTO with respect to mortality or morbidity, either because the RVOTO was not serious (25-50 mmHg) or because the number of patients with RVOTO was too small for there to be a detectable difference in the above parameters between the two groups.

Impaired cardiac function may be associated with the high mortality or morbidity noted among HCM patients with SRVH. In our study, an NYHA class of III or worse was an independent predictor of cardiovascular death, and 25% patients suffered from progressive heart failure that increased to NYHA class III or worse during the follow-up period. RV involvement was correlated with worsening NYHA functional class status [19]. In HCM patients with SRVH, edema was observed in 20.6% of patients, and NT-proBNP levels were significantly elevated compared with those in ApHCM patients. Both edema and elevated NT-proBNP levels may be indicative of RV dysfunction [22]. Moreover, myocardial fibrosis or scarring detected using CMR and LGE has been associated with increased ventricular stiffness and reduced ventricular compliance, which lead to impaired ventricular relaxation, elevated end-diastolic pressure, and, consequently, heart failure [23]. The high incidence of LGE involving the RV wall may be another indicator of impaired RV function. RV dysfunction in HCM has been independently linked to both death and heart transplantation [16], and impaired RV diastolic function is strongly correlated with death secondary to heart failure [24].

### Dominance of the right ventricle

Pulmonary hypertension may induce mild RV hypertrophy during its early stages, but it may also lead to rapid RV thinning and dilation [25]. The right ventricle may be the primary and predominant chamber affected in HCM [26]. Of our patients, one patient exhibited an isolated RV wall thickness of 17 mm (the maximum left ventricular wall thickness was 13 mm), demonstrating that the right ventricle alone can be affected in HCM [27–28]. According to the “spill-over effect” theory [8], mild left ventricular hypertrophy may occur secondary to severe RV hypertrophy. Additionally, the typical pathological changes of HCM involving the RV wall were of sufficient severity to be detected via histopathology and CMR, whereas the changes affecting the left ventricular wall were mild. Lastly, RV outflow obstruction was noted, whereas the hemodynamics of the left ventricular outflow tract were normal.

### Genetic background

More than 27 genes and 1,400 locations linked to HCM have been described [29]. Mutations in known sarcomere protein genes were previously detected in approximately 35% to 65% of HCM patients [30]. However, 90% of HCM patients with SRVH were found to possess relevant sarcomere protein mutations when the WGS technique was utilized in this study. Following data filtering and based on the 1000 Genomes Project dataset, the NHLBI exome sequencing project database, DbSNP 131, the International HapMap Project dataset, and data generated using prediction software, we determined that mutations involving sarcomere proteins may be related to HCM.

*MYBPC3* gene mutations have previously been described in two patients with RV hypertrophy [31–32]. Variations in the *MYH7* and *TTN* genes were more common among patients with RV hypertrophy in this study, followed by variations in *MYBPC3*. There did not appear to be a relationship between genotype and severe RV hypertrophy in our study; however, two or more gene mutations may result in multiple morphological changes, earlier disease onset and poorer outcomes compared with single mutations [33]. Patients with more than one HCM-causing mutation may be at an increased risk for SCD, left ventricular dilation, severe heart failure, and premature death [33]. In our study population, 73% of HCM patients with SRVH carried two or more sarcomere gene mutations; thus, it may be inferred that the relatively poor prognosis associated with this phenotype may be associated with having multiple gene mutations.

We also identified variants pertaining to other cardiomyopathies and ion-channel diseases, the majority of which were of unknown importance. We found that these particular individual variants occurred at a frequency of less than 0.5% in the 1000 Genomes Project dataset, which suggested that they may modulate the HCM phenotype [34]. It is difficult to define the relationships between these variants and the pathogenesis of HCM, as most of the patients possessing these mutations also had at least one sarcomere gene mutation, but some mutations in RYR2, ANK2, CAV3 and SCN5A have been shown to be pathological [34]. Moreover, it may be inferred that the mutations associated with ARVC and ion-channel diseases (identified in our study) are potential phenotype modifiers in HCM patients with SRVH and may thus effect the prognoses and the heterogeneity of HCM [34]. Additional functional studies of genetic data with WGS are needed.

### Limitations

Our study had several limitations. This study involved retrospectively enrolled HCM patients. Its design may thus have been flawed, and the data pertaining to some variables may have been incomplete. Studies including more patients with SRVH are needed to obtain a more accurate understanding of this phenotype. Additionally, CMR and blood samples were not available for all patients who enrolled early in the study period. Moreover, because of the small sample size of the study, we were unable to perform a semi-quantitative analysis that may have enabled us to quantify the degree of myocyte hypertrophy. Electron microscopy and TUNEL staining would also have been helpful histological strategies for demonstrating the pathological changes associated with HCM. Finally, in addition to differences in morphology, complex factors may also explain the various clinical features and prognosis of HCM. More studies are needed to understand these aspects of the disease.

## Conclusion

SRVH is an uncommon phenotype in HCM and is characterized by progressive clinical deterioration and a relatively poor prognosis. Multiple gene mutations are associated with this phenotype and may be related to the mechanisms underlying the prognosis and heterogeneity associated with HCM.

## Supporting information

S1 Fig Variant filtration flow chart for WGS of HCM patients with SRVH. This figure shows the stepwise variant filtration process for evaluating possible pathogenic mutations. ARVC, arrhythmogenic RV cardiomyopathy; BR, Brugada syndrome; DbSNP, Single Nucleotide Polymorphism Database; DCM, dilated cardiomyopathy; ESP 6500, NHLBI Grand Opportunity Exome Sequencing Project; LQTS, long-QT syndrome; RCM, restrictive cardiomyopathy; SQTS, short-QT syndrome.
(TIF)

S1 File Study data of HCM patients with SRVH and patients with ApHCM. This file shows the original data of all patients in this study.
(CSV)
