# Supplementary figures and images for: The clinical features, outcomes and genetic characteristics of hypertrophic cardiomyopathy patients with severe right ventricular hypertrophy
Source: PLoS One. 2017 Mar 21;12(3):e0174118. doi: 10.1371/journal.pone.0174118 (PMC5360271; doi:10.1371/journal.pone.0174118)

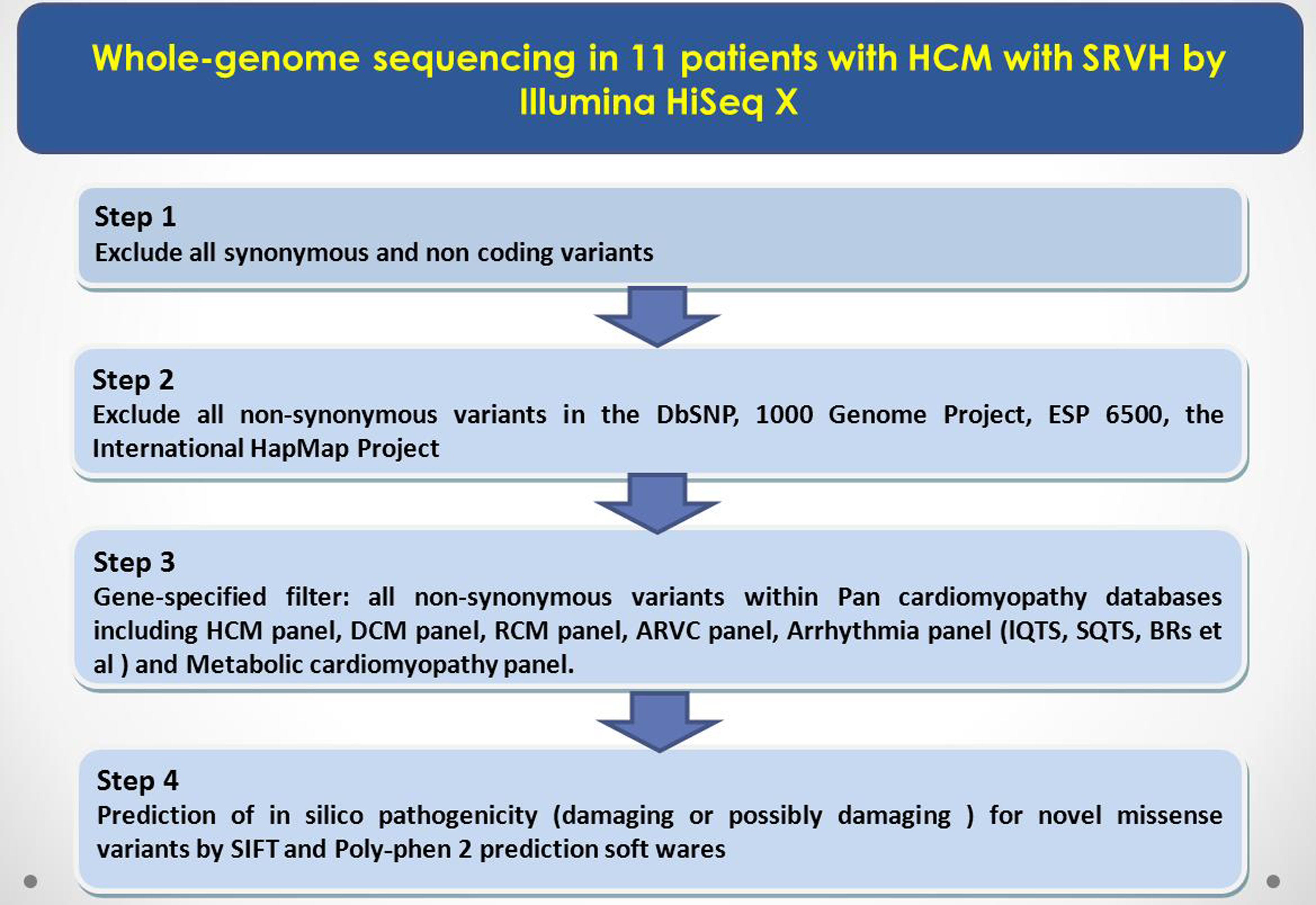

Supplement: S1 Fig — This figure shows the stepwise variant filtration process for evaluating possible pathogenic mutations. ARVC, arrhythmogenic RV cardiomyopathy; BR, Brugada syndrome; DbSNP, Single Nucleotide Polymorphism Database; DCM, dilated cardiomyopathy; ESP 6500, NHLBI Grand Opportunity Exome Sequencing Project; LQTS, long-QT syndrome; RCM, restrictive cardiomyopathy; SQTS, short-QT syndrome. (TIF) [file pone.0174118.s001.tif]
